# The chromosomal genome sequence of the stone sponge *Petrosia ficiformis *(Poiret, 1789) and its associated microbial metagenome sequences

**DOI:** 10.12688/wellcomeopenres.24743.1

**Published:** 2025-08-18

**Authors:** Laura Steindler, Manuel Maldonado, Lucia Pita, Ana Riesgo, Dirk Erpenbeck, Ute Hentschel, Graeme Oatley, Elizabeth Sinclair, Eerik Aunin, Noah Gettle, Camilla Santos, Michael Paulini, Haoyu Niu, Victoria McKenna, Rebecca O’Brien, Wellcome Sanger Institute Tree of Life Management, Samples and Laboratory Team, Wellcome Sanger Institute Scientific Operations: Sequencing Operations, Wellcome Sanger Institute Tree of Life Core Informatics Team, EBI Aquatic Symbiosis Genomics Data Portal Team, Aquatic Symbiosis Genomics Project Leadership

**Affiliations:** 1 Department of Marine Biology, Leon H. Charney School of Marine Sciences, University of Haifa, Haifa, Israel; 2 Department of Marine Ecology, Center for Advanced Studies of Blanes (CEAB-CSIC), Girona, Spain; 3 Institute of Marine Sciences – CSIC, Barcelona, Spain; 4 Integrated Marine Ecology group, Institute of Marine Research IIM-CSIC, Vigo, Spain; 5 Department of Biodiversity and Evolutionary Biology, MNCN-National Museum of Natural Sciences, Madrid, Spain; 6 Department of Earth and Environmental Sciences, Ludwig-Maximilian University of Munich, Munich, Germany; 7 RU Marine Symbioses, GEOMAR Helmholtz Centre for Ocean Research Kiel, Kiel, Germany; 8 Tree of Life, Wellcome Sanger Institute, Hinxton, England, UK

**Keywords:** Petrosia ficiformis, stone sponge, genome sequence, chromosomal, Haplosclerida

## Abstract

We present a genome assembly from an individual *Petrosia ficiformis* (stone sponge; Porifera; Demospongiae; Haplosclerida; Petrosiidae). The genome sequence is 191.3 megabases in span. Most of the assembly is scaffolded into 18 chromosomal pseudomolecules. The mitochondrial genome has also been assembled and is 18.89 kilobases in length. Gene annotation of the host organism assembly identified 18,339 protein coding genes. The metagenome of the specimen was also assembled, and 112 binned bacterial genomes were identified, including 57 high-quality MAGs. Besides MAGs characteristic of HMA sponge symbionts (i.e., Chloroflexota, Acidobacteriota), the *P. ficiformis* specific symbiont *Candidatus* Synechococcus feldmanni (formerly *Aphanocapsa feldmanni* (Cyanobacteriota) was recovered, as well as notably MAGs of several candidate phyla ( *Candidatus* Latescibacteria, Poribacteria, Tectomicrobia, Dadabacteria, Kapabacteria and Binatia).

## Species taxonomy: *Petrosia ficiformis*

Eukaryota; Opisthokonta; Metazoa; Porifera; Demospongiae; Heteroscleromorpha; Haplosclerida; Petrosiidae; *Petrosia* (Poiret, 1789) (NCBI:txid68564).

## Background

*Petrosia ficiformis* (Poiret, 1789), informally called ‘the stony sponge’, is a species widely distributed in the Mediterranean Sea and the Eastern Atlantic Ocean, at depths ranging from 3 to 100 metres ( Van Soest *et al.*, 2017). In the sublittoral, it grows preferentially on vertical walls, overhangs and in cave entrances, always attached to hard substrates ( Sarà *et al.*, 1998). Its body can be a flattened subsphere, sometimes lobate, with anastomosing lobules. The distinctive red-wine colour of this species results from the presence of symbiotic cyanobacteria inhabiting extracellularly in the subdermal mesohyl of the body areas exposed to light. In contrast, shaded body parts or entire individuals growing in caves are white due to loss of cyanobacteria ( Wilkinson & Vacelet, 1979). These cyanobacteria are not obligate symbionts, since they are not vertically transmitted, but apparently acquired from the seawater by juveniles and adults ( Maldonado & Riesgo, 2009). The cyanobacteria of adults may also be lost as result of environmental stress, wherein the depigmented sponge’s surface can undergo necrosis ( Cerrano *et al.*, 2001).

*P. ficiformis* is a gonochoristic species (i.e., separate sexes), with no macroscopic sexual dimorphism distinguishing males from females. As far as is known, the species only reproduces once a year. Oogenesis requires several months to complete oocyte maturation, while spermatogenesis develops rapidly, with spermatic cysts found in the mesohyl of males for no longer than 2.5 weeks. This species is oviparous, releasing oocytes and sperm cells into the water column for external fertilisation ( Lepore *et al.*, 1995; Maldonado & Riesgo, 2009). In the Spanish Mediterranean spawning occurs in late autumn, typically in early December. The zygotes resulting from the external fertilisation in laboratory conditions develop into ciliated crawling larvae, which appear unable to swim and, therefore, are assumed to have limited dispersal potential ( Maldonado & Riesgo, 2009). This poor dispersal likely contributes to the restricted gene flow detected between *P. ficiformis* populations ( Riesgo *et al.*, 2019). In contrast to other sponge species, the population effectives of *P. ficiformis* appear to not rely on asexual reproduction ( Riesgo *et al.*, 2019).

*P. ficiformis* is a high microbial abundance (HMA) sponge harbouring a dense and morphologically varied community of microorganisms, mostly housed by specialised cells known as bacteriocytes ( Vacelet & Donadey, 1977). Yet, the *P. ficiformis*’ bacteriocyte, known as pocket bacteriocyte, is unique in Porifera. Unlike the bacteriocytes of other sponges, it hosts microbes extracellularly rather than intracellularly. To do this, the cells fold in on themselves to form a pocket-like extracellular cavity in which microbes are housed ( Carrier *et al.*, 2022; Maldonado, 2007). The taxonomically diverse symbiont community comprises members of Cyanobacteriota, Chloroflexota, Gammaproteobacteria, Acidobacteriota, and Archaea, among others ( Burgsdorf *et al.*, 2014). The metagenome-assembled genomes (MAGs) of several symbionts of *P. ficiformis* were described in detail for members of the Archaea ( Haber *et al.*, 2021), Verrucomicrobiota ( Sizikov *et al.*, 2020), and Cyanobacteriota ( Burgsdorf *et al.*, 2019). The latter belonging to the species *Candidatus* Synechococcus feldmanni, formerly called *Aphanocapsa feldmanni* ( Burgsdorf *et al.*, 2019; Usher *et al.*, 2004). *Ca.* S. feldmanni differs from the congeneric symbiont *Candidatus* Synechococcus spongiarum in the following aspects: (i) *Ca.* S. feldmanni is specific to *P. ficiformis,* while *Ca.* S. spongiarum is widely distributed among sponges; (ii) *Ca.* S. feldmanni is located intracellularly, while *Ca.* S. spongiarum, extracellularly; (iii) the contribution of fixed carbon in *Ca.* S. feldmanni to its host sponge is minimal compared to that of *Ca.* S. spongiarum ( Burgsdorf *et al.*, 2022), and (iv) *Ca.* S. feldmanni is acquired horizontally ( Britstein *et al.*, 2020) while *Ca.* S. spongiarum is transferred vertically via “leaky” transmission ( Webster *et al.*, 2010). The absence of microbes within spermatozoa, oocytes, embryos and larvae suggests that each new generation of *P. ficiformis* acquires most microbes from the surrounding sea water ( Lepore *et al.*, 1995; Maldonado, 2007; Maldonado & Riesgo, 2009).

The genus *Petrosia* is recognised as a rich source of bioactive metabolites of which some may be of microbial origin ( Lee *et al.*, 2021). Characteristic secondary metabolites of the genus *Petrosia*, isolated both from *P. ficiformis* and its predator nudibranch *Peltodoris atromaculata,* are high-molecular weight polyacetylenes, termed petroformynes, which act as a potent toxin against *Artemia salina* and which inhibit sea urchin egg development ( Castiello *et al.*, 1980; Guo *et al.*, 1994). Other typical secondary metabolites found in the genus *Petrosia* are sterols, including Petrosterol ( Sica & Zollo, 1978).

Sponges play an important role in the cycling of some organic and inorganic marine nutrients. One of these elements is silicon (Si), which sponge cells take up in the form of silicic acid dissolved in the seawater and polymerise it into solid biogenic silica, forming the structural parts (named spicules) that compose the sponge’s skeleton. Interestingly, *P. ficiformis* is one of the most heavily skeletonized sponge species in the Mediterranean sublittoral and their siliceous spicules are highly resistant to dissolution after sponge death ( Maldonado *et al.*, 2005). Therefore, the populations of *P. ficiformis* are though to become significant local sinks of Si, through the burial of its spicules in the marine sediments ( Maldonado *et al.*, 2019). The abundance and the ecological role of this species are somehow threatened because the populations suffer periodically events of mass mortality related to heat waves ( Garrabou *et al.*, 2009), but the exact mechanisms that cause sponge death remain unclear.

The biotechnological potential of *P. ficiformis* has been explored extensively ( Cerrano *et al.*, 2022). For example, the round-shaped 3D sponge cell aggregates with telomerase activity are useful for diverse investigations of sponge cell biology ( Pozzolini *et al.*, 2004; Valisano *et al.*, 2012).

## Genome sequence report

### Sequencing data

**Figure 1.**
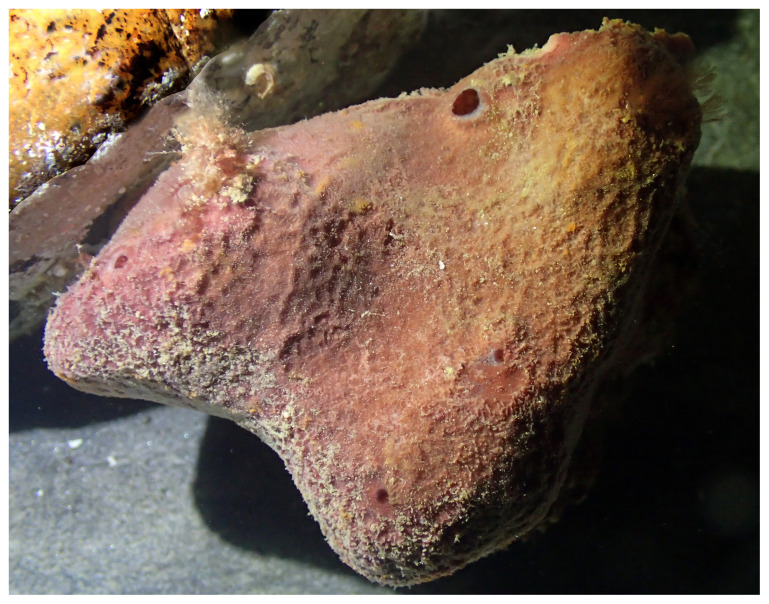
Photograph of the *Petrosia ficiformis* (odPetFici3) specimen from which samples were taken for genome sequencing.

The genome of a specimen of *Petrosia ficiformis* ( Figure 1) was sequenced using Pacific Biosciences single-molecule HiFi long reads, generating 89.01 Gb (gigabases) from 8.46 million reads. Based on this estimated genome size, the sequencing data provided approximately 25× coverage of the genome. Chromosome conformation Hi-C data produced 36.92 Gb from 244.52 million reads. Table 1 summarises the specimen and sequencing information.

**Table 1.** Specimen and sequencing data for *Petrosia ficiformis*.

Project information			
**Study title**	*Petrosia ficiformis*		
**Umbrella BioProject**	PRJEB56076		
**Species**	*Petrosia ficiformis*		
**BioSpecimen**	SAMEA9361899		
**NCBI taxonomy ID**	68564		
Specimen information			
**Technology**	**ToLID**	**BioSample accession**	**Organism part**
**PacBio long read sequencing**	odPetFici3	SAMEA9361945	Somatic tissue
**Hi-C sequencing**	odPetFici3	SAMEA9361935	Somatic tissue
**RNA sequencing**	odPetFici3	SAMEA9361947	Somatic tissue
Sequencing information			
**Platform**	**Run accession**	**Read count**	**Base count (Gb)**
**Hi-C Illumina NovaSeq 6000**	ERR10297827	6.10e+07	9.22
**Hi-C Illumina NovaSeq 6000**	ERR10297828	1.83e+08	27.71
**PacBio Sequel IIe**	ERR10224933	6.06e+06	72.37
**PacBio Sequel IIe**	ERR10224934	2.51e+05	0.83
**PacBio Sequel IIe**	ERR10224935	2.15e+06	15.82
**RNA Illumina NovaSeq 6000**	ERR10378034	6.46e+07	9.75

### Host assembly statistics

The primary haplotype was assembled, and contigs corresponding to an alternate haplotype were also deposited in INSDC databases. The assembly was improved by manual curation, which corrected 37 misjoins or missing joins and removed 13 haplotypic duplications. These interventions reduced the total assembly length by 5.47%, decreased the scaffold count by 21.74%, and increased the scaffold N50 by 16.62%. The final assembly has a total length of 191.25 Mb in 35 scaffolds, and a scaffold N50 of 9.94 Mb ( Table 2).

**Table 2.** Genome assembly data for *Petrosia ficiformis*.

Genome assembly	
Assembly name	odPetFici3.1
Assembly accession	GCA_947044365.1
*Alternate haplotype accession*	*GCA_947044245.1*
Assembly level for primary assembly	chromosome
Span (Mb)	191.25
Number of contigs	110
Number of scaffolds	35
Longest scaffold (Mb)	63.28
Assembly metric	Measure
Contig N50 length	3.95 Mb
Scaffold N50 length	9.94 Mb
Consensus quality (QV)	Primary: 62.8; alternate: 52.6; combined 55.1
BUSCO *	C:82.3%[S:81.4%,D:0.8%],F:6.5%,M:11.2%,n:954
Percentage assembled	99.91%
Organelles	Mitochondrial genome: 18.89 kb

* BUSCO scores based on the metazoa_odb10 BUSCO set using version 5.3.2. C = complete [S = single copy, D = duplicated], F = fragmented, M = missing, n = number of orthologues in comparison.

**Figure 2.**
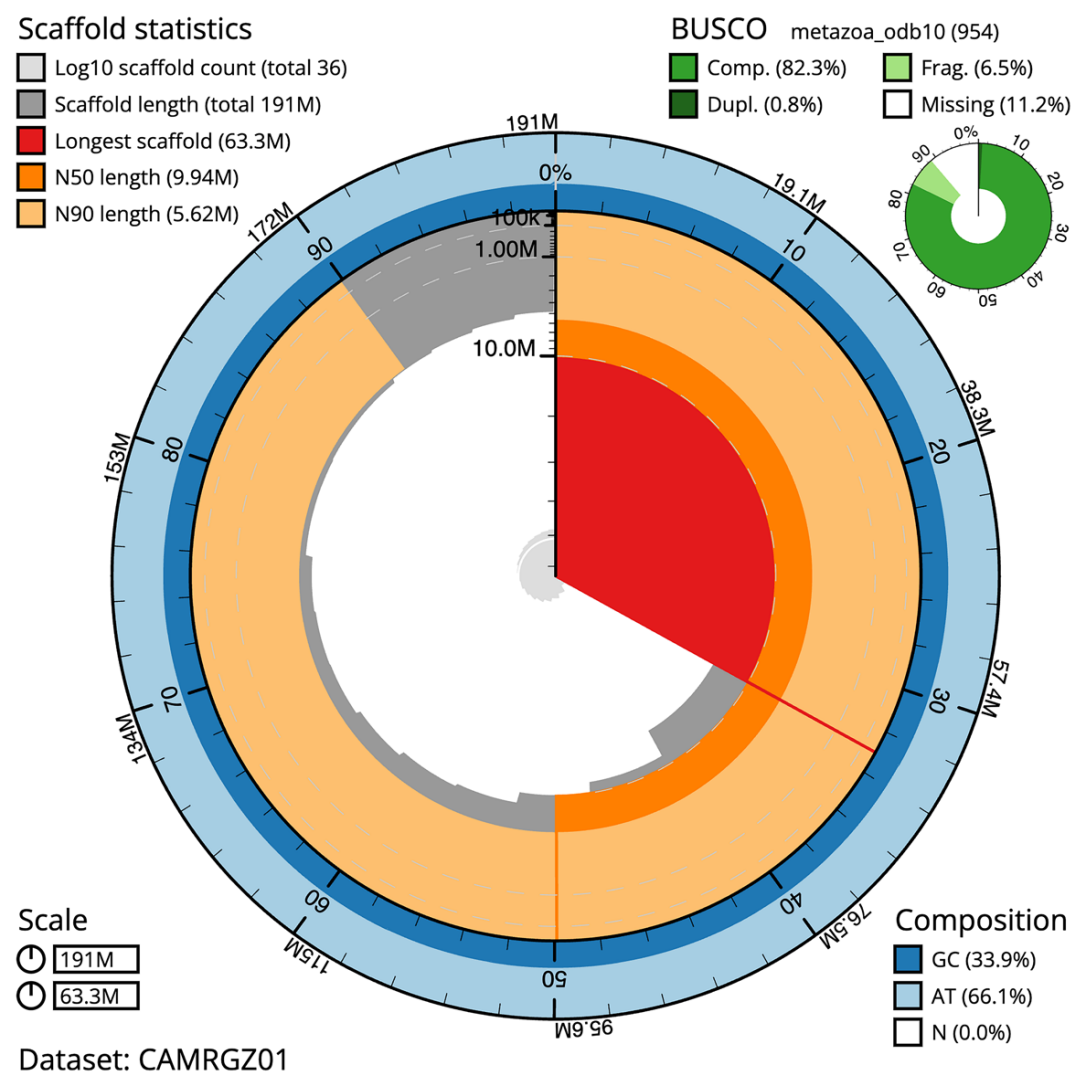
Genome assembly of *Petrosia ficiformis*, odPetFici3.1: metrics. The BlobToolKit Snailplot shows N50 metrics and BUSCO gene completeness. The main plot is divided into 1,000 size-ordered bins around the circumference with each bin representing 0.1% of the 191,273,811 bp assembly. The distribution of scaffold lengths is shown in dark grey with the plot radius scaled to the longest scaffold present in the assembly (63,279,122 bp, shown in red). Orange and pale-orange arcs show the N50 and N90 scaffold lengths (9,942,894 and 5,615,395 bp), respectively. The pale grey spiral shows the cumulative scaffold count on a log scale with white scale lines showing successive orders of magnitude. The blue and pale-blue area around the outside of the plot shows the distribution of GC, AT and N percentages in the same bins as the inner plot. A summary of complete, fragmented, duplicated and missing BUSCO genes in the metazoa_odb10 set is shown in the top right. An interactive version of this figure is available at https://blobtoolkit.genomehubs.org/view/CAMRGZ01/dataset/CAMRGZ01/snail.

**Figure 3.**
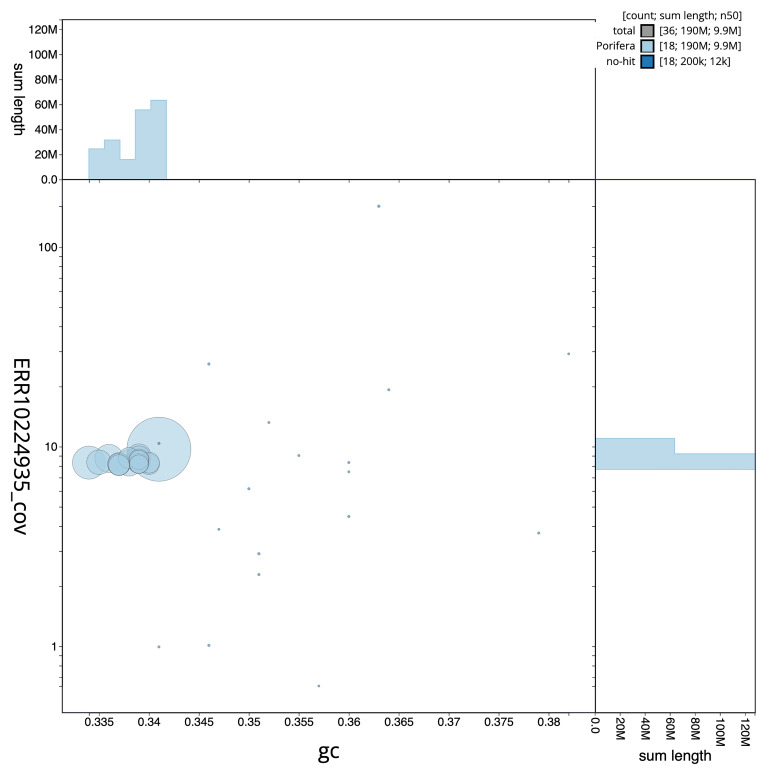
Genome assembly of *Petrosia ficiformis*, odPetFici3.1: BlobToolKit GC-coverage plot. Scaffolds are coloured by phylum. Circles are sized in proportion to scaffold length. Histograms show the distribution of scaffold length sum along each axis. An interactive version of this figure is available at https://blobtoolkit.genomehubs.org/view/CAMRGZ01/dataset/CAMRGZ01/blob.

**Figure 4.**
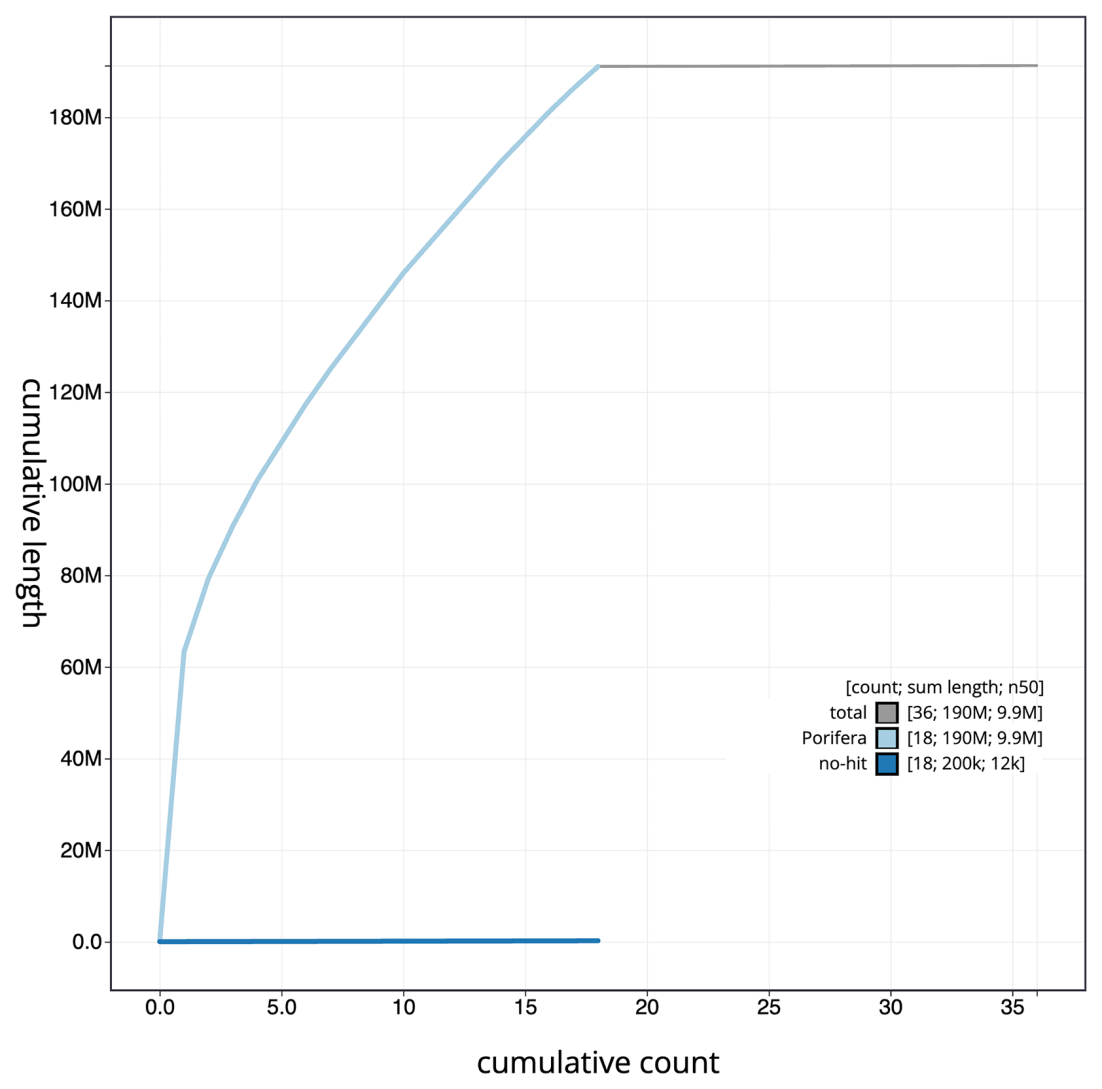
Genome assembly of *Petrosia ficiformis*, odPetFici3.1: BlobToolKit cumulative sequence plot. The grey line shows cumulative length for all scaffolds. Coloured lines show cumulative lengths of scaffolds assigned to each phylum using the buscogenes taxrule. An interactive version of this figure is available at https://blobtoolkit.genomehubs.org/view/CAMRGZ01/dataset/CAMRGZ01/cumulative.

The snail plot in Figure 2 provides a summary of the assembly statistics, indicating the distribution of scaffold lengths and other assembly metrics. Figure 3 shows the distribution of scaffolds by GC proportion and coverage. Figure 4 presents a cumulative assembly plot, with separate curves representing different scaffold subsets assigned to various phyla, illustrating the completeness of the assembly.

**Figure 5.**
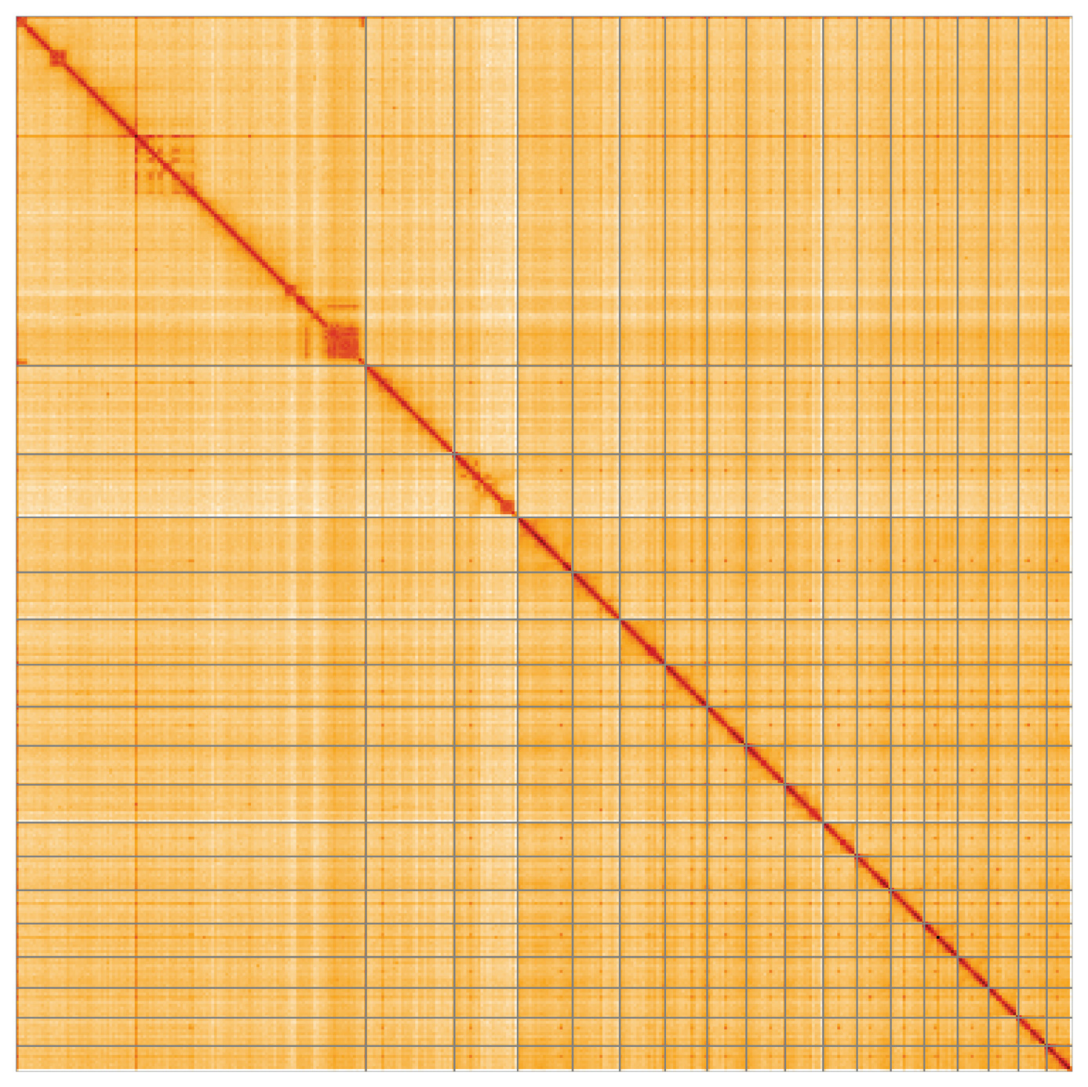
Genome assembly of *Petrosia ficiformis*, odPetFici3.1: Hi-C contact map of the odPetFici3.1 assembly, visualised using HiGlass. Chromosomes are shown in order of size from left to right and top to bottom. An interactive version of this figure may be viewed at https://genome-note-higlass.tol.sanger.ac.uk/l/?d=Nyhm_TNaQMugJ1TXa66S_A.

Most of the assembly sequence (99.91%) was assigned to 18 chromosomal-level scaffolds. These chromosome-level scaffolds, confirmed by Hi-C data, are named according to size ( Figure 5; Table 3).

**Table 3.** Chromosomal pseudomolecules in the genome assembly of *Petrosia ficiformis*, odPetFici3.

INSDC accession	Name	Length (Mb)	GC%
OX345636.1	1	63.28	34
OX345637.1	2	16.0	33.5
OX345638.1	3	11.43	33.5
OX345639.1	4	9.94	34
OX345640.1	5	8.53	33.5
OX345641.1	6	8.19	34
OX345642.1	7	7.59	34
OX345643.1	8	7.09	33.5
OX345644.1	9	7.02	33.5
OX345645.1	10	6.87	34
OX345646.1	11	6.12	34
OX345647.1	12	6.11	34
OX345648.1	13	6.03	33.5
OX345649.1	14	6.02	34
OX345650.1	15	5.62	34
OX345651.1	16	5.39	34
OX345652.1	17	5.11	34
OX345653.1	18	4.74	34
OX345654.1	MT	0.02	36.5

The mitochondrial genome was also assembled. This sequence is included as a contig in the multifasta file of the genome submission and as a standalone record in GenBank.

### Host assembly quality metrics

The primary haplotype has a QV of 62.8, and the combined primary and alternate assemblies achieve an estimated QV of 55.1. The estimated Quality Value (QV) of the final host assembly is 62.8, and the assembly has a BUSCO v5.3.2 completeness of 82.3% (single = 81.4%, duplicated = 0.8%), using the metazoa_odb10 reference set ( *n* = 954).

### Taxonomic validation

*Petrosia ficiformis* is reported from many habitats in the Mediterranean, and in several morphotypes, which implies the presence of a species complex (see, e.g. Burgsdorf *et al.*, 2014). Sequences of type material have not been available for taxonomic validation. However, the mitochondrial COI (Folmer region) is identical to *Petrosia ficiformis* reference material provided from NCB Naturalis, Leiden, identified by Nicole de Voogd (SNSB-BSPG.GW35178), and identical to the “violet morph” in Burgsdorf *et al.*, 2014.

## Metagenome report

**Figure 6.**
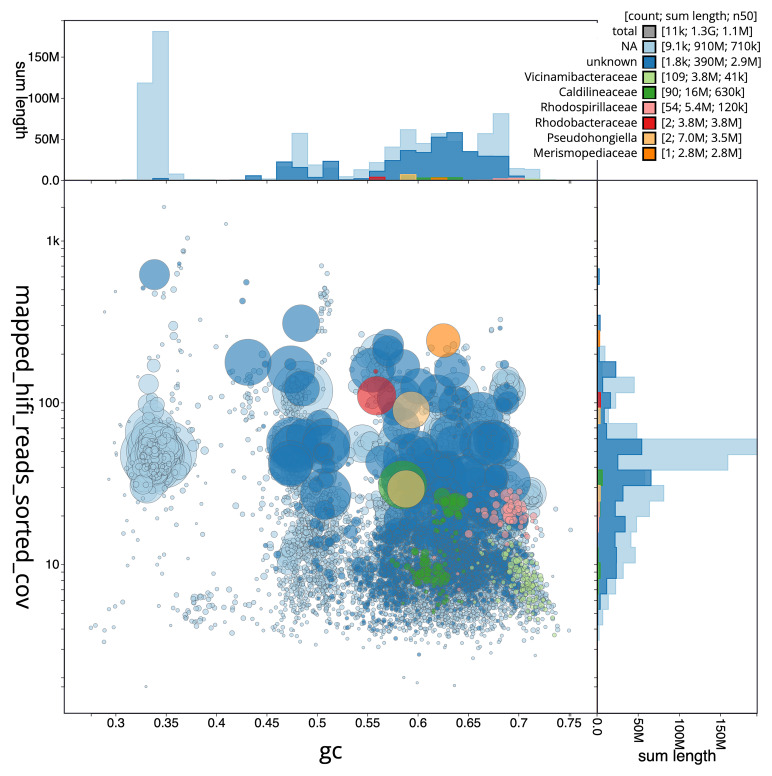
Blob plot of base coverage in mapped against GC proportion for sequences in the *Petrosia ficiformis* metagenome. Binned contigs are coloured by family. Circles are sized in proportion to sequence length on a square root scale, ranging from 1,919 to 10,494,744. Histograms show the distribution of sequence length sum along each axis. An interactive version may be viewed here.

**Figure 7.**
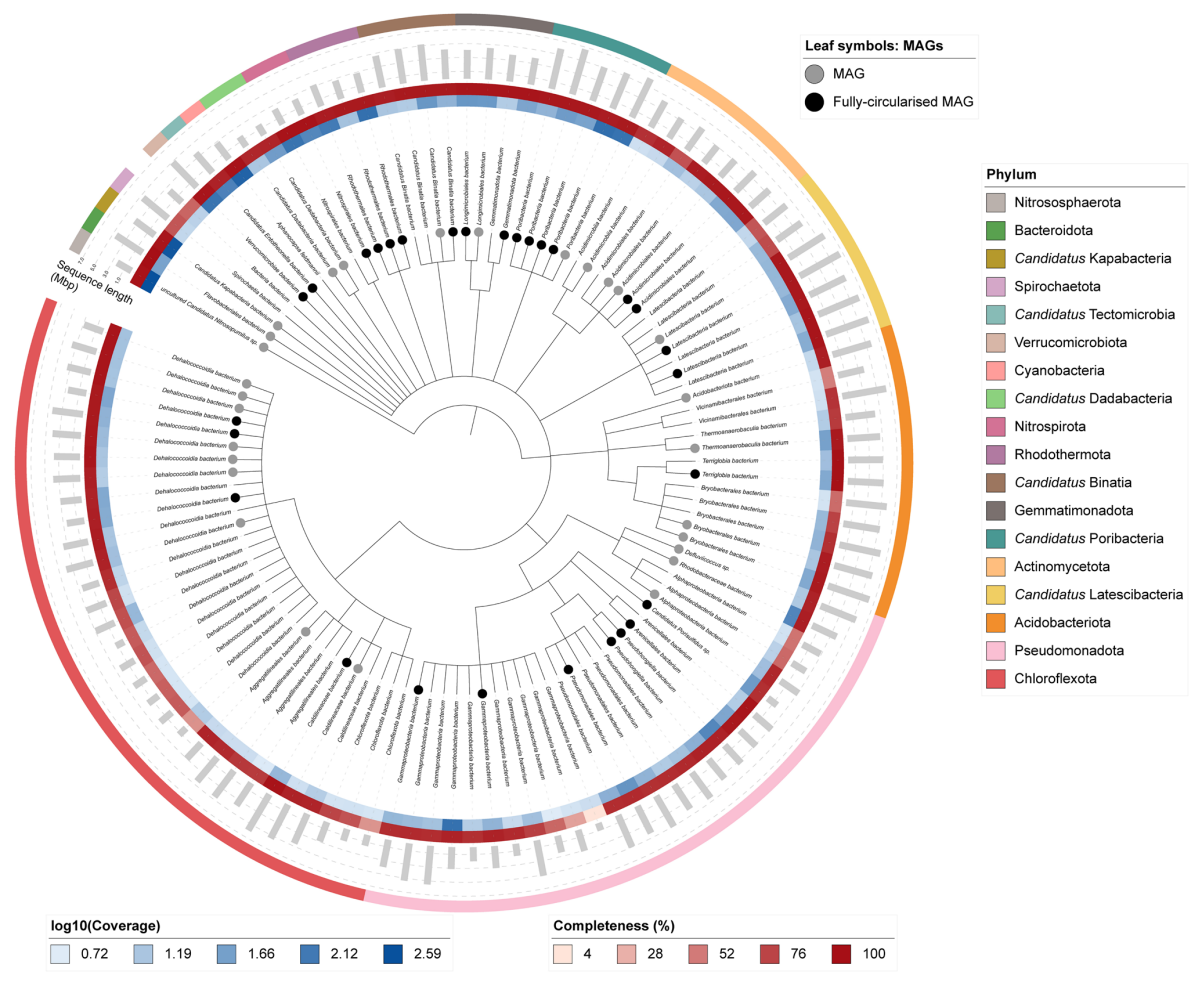
Cladogram showing the taxonomic placement of metagenome bins, constructed using NCBI taxonomic identifiers with *taxonomizr* and annotated in iTOL. Colours indicate phylum-level taxonomy. Additional tracks show sequencing coverage (log _10_), genome size (Mbp), and completeness. Bins that meet the criteria for MAGs are marked with a grey circle and fully circularised MAGs are marked in black.

One hundred and twelve binned genomes were generated from the metagenome assembly ( Figure 6) of which 57 were classified as high-quality metagenome assembled genomes (MAGs) (see methods). The completeness values for these binned genomes range from approximately 40% to 99% with contamination below 10%. The full set of bins are available on FigShare ( https://doi.org/10.6084/m9.figshare.28749905). A cladogram is shown in Figure 7.

### Host genome annotation

The *Petrosia ficiformis* annotation performed at the Wellcome Sanger Institute using BRAKER3 produced 18,339 genes, with an average gene length of 5,157 bases, with an average of 7.4 exons per transcript. This annotation is provided as an UCSC assembly hub and raw downloads at https://github.com/Aquatic-Symbiosis-Genomics-Project/sponge_annotations/tree/main/results/odPetFici3.

## Methods

### Sample acquisition

A specimen of *Petrosia ficiformis* (specimen ID GHC0000184, ToLID odPetFici3) was collected from Blanes, Girona, Spain (latitude 41.67, longitude 2.80) on 2021-02-01 by SCUBA diving. The specimen was collected and identified by Manuel Maldonado (CEAB-CSIC) and preserved by snap-freezing.

### Nucleic acid extraction

The workflow for high molecular weight (HMW) DNA extraction at the Wellcome Sanger Institute (WSI) includes a sequence of protocols for sample preparation, sample homogenisation, DNA extraction, fragmentation and clean-up. Protocols developed by the WSI Tree of Life laboratory are publicly available on protocols.io ( Denton *et al.*, 2023). In sample preparation, the odPetFici3 sample was weighed and dissected on dry ice ( Jay *et al.*, 2023). Prior to DNA extraction, the sponge sample was bathed in “L buffer” (10 mM Tris, pH 7.6, 100 mM EDTA, 20 mM NaCl), minced into small pieces using a scalpel and the cellular interior separated from the mesohyl using forceps ( Lopez, 2022). HMW DNA was extracted using the Manual MagAttract v1 protocol ( Strickland *et al.*, 2023b). DNA was sheared into an average fragment size of 12–20 kb in a Megaruptor 3 system ( Todorovic *et al.*, 2023). Sheared DNA was purified by solid-phase reversible immobilisation ( Strickland *et al.*, 2023a), using AMPure PB beads to eliminate shorter fragments and concentrate the DNA. The concentration of the sheared and purified DNA was assessed using a Nanodrop spectrophotometer and Qubit Fluorometer and Qubit dsDNA High Sensitivity Assay kit. Fragment size distribution was evaluated by running the sample on the FemtoPulse system.

### Sequencing

Pacific Biosciences HiFi circular consensus DNA sequencing libraries were constructed according to the manufacturers’ instructions. Poly(A) RNA-Seq libraries were constructed using the NEB Ultra II RNA Library Prep kit. DNA and RNA sequencing was performed by the Scientific Operations core at the WSI on Pacific Biosciences SEQUEL II (HiFi) and Illumina NovaSeq 6000 (RNA-Seq) instruments. Hi-C data were also generated from tissue of odPetFici3 using the Arima2 kit and sequenced on the Illumina NovaSeq 6000 instrument.

### Genome assembly, curation and evaluation

***Host genome assembly***

Assembly was carried out with Hifiasm ( Cheng *et al.*, 2021) and haplotypic duplications were identified and removed with purge_dups ( Guan *et al.*, 2020). The assembly was then scaffolded with Hi-C data ( Rao *et al.*, 2014) using YaHS ( Zhou *et al.*, 2023). The assembly was checked for contamination and corrected using the gEVAL system ( Chow *et al.*, 2016) as described previously ( Howe *et al.*, 2021). Manual curation was performed using gEVAL, HiGlass ( Kerpedjiev *et al.*, 2018) and Pretext ( Harry, 2022). The mitochondrial genome was assembled using MitoHiFi ( Uliano-Silva *et al.*, 2023), which runs MitoFinder ( Allio *et al.*, 2020) and uses these annotations to select the final mitochondrial contig and to ensure the general quality of the sequence.

### Taxonomic verification

*Petrosia ficiformis* is reported from many habitats in the Mediterranean, and in several morphotypes, which implies the presence of a species complex (see, for example, Burgsdorf *et al.*, 2014). Sequences of type material have not been available for taxonomic validation. However, the mitochondrial CO1 (Folmer region) is identical to *Petrosia ficiformis* reference material provided from NCB Naturalis, Leiden, identified by Nicole de Voogd (SNSB-BSPG.GW35178), and identical to the “violet morph” in Burgsdorf *et al*. (2014).

***Host assembly quality assessment***

The Merqury.FK tool ( Rhie *et al.*, 2020), run in a Singularity container ( Kurtzer *et al.*, 2017), was used to evaluate assembly quality for the primary and alternate haplotypes using the *k*-mer databases ( *k* = 31) that were computed prior to genome assembly. The analysis outputs included assembly QV scores.

A Hi-C contact map was produced for the final version of the assembly. The Hi-C reads were aligned using bwa-mem2 ( Vasimuddin *et al.*, 2019) and the alignment files were combined using SAMtools ( Danecek *et al.*, 2021). The Hi-C alignments were converted into a contact map using BEDTools ( Quinlan & Hall, 2010) and the Cooler tool suite ( Abdennur & Mirny, 2020). The contact map is visualised in HiGlass ( Kerpedjiev *et al.*, 2018).

The genome was also analysed within the BlobToolKit environment ( Challis *et al.*, 2020) and BUSCO scores ( Manni *et al.*, 2021) were calculated.

***Metagenome assembly***

The metagenome assembly was generated using metaMDBG ( Benoit *et al.*, 2024) and binned using MetaBAT2 ( Kang *et al.*, 2019), MaxBin ( Wu *et al.*, 2014), bin3C ( DeMaere & Darling, 2019), and MetaTOR. The resulting bin sets of each binning algorithm were optimised and refined using MAGScoT ( Rühlemann *et al.*, 2022). PROKKA ( Seemann, 2014) was used to identify tRNAs and rRNAs in each bin, CheckM ( Parks *et al.*, 2015) (checkM_DB release 2015-01-16) was used to assess bin completeness/contamination, and GTDB-TK ( Chaumeil *et al.*, 2022) (GTDB release 214) was used to taxonomically classify bins. Taxonomic replicate bins were identified using dRep ( Olm *et al.*, 2017) with default settings (95% ANI threshold). The final bin set was filtered for bacteria and archaea. All bins were assessed for quality and categorised as metagenome-assembled genomes (MAGs) if they met the following criteria: contamination ≤ 5%, presence of 5S, 16S, and 23S rRNA genes, at least 18 unique tRNAs, and either ≥ 90% completeness or ≥ 50% completeness with fully circularised chromosomes. Bins that did not meet these thresholds, or were identified as taxonomic replicates of MAGs, were retained as ‘binned metagenomes’ provided they had ≥ 50% completeness and ≤ 10% contamination. A cladogram based on NCBI taxonomic assignments was generated using the ‘taxonomizr’ package in R. The tree was visualised and annotated using iTOL ( Letunic & Bork, 2024). Software tool versions and sources are given in Table 4.

**Table 4.** Software tools: versions and sources.

Software tool	Version	Source
BEDTools	2.30.0	https://github.com.com/arq5x/bedtools2
bin3C	0.3.3	https://github.com.com/cerebis/bin3C
BlobToolKit	4.2.1	https://github.com.com/blobtoolkit/blobtoolkit
BUSCO	5.3.2	https://gitlab.com/ezlab/busco
CheckM	1.2.1	https://github.com.com/Ecogenomics/CheckM
Cooler	0.8.11	https://github.com.com/open2c/cooler
dRep	3.4.0	https://github.com.com/MrOlm/drep
FastK	427104ea91c78c3b8b8b49f1a7d6bbeaa869ba1c	https://github.com.com/thegenemyers/FASTK
gEVAL	-	https://geval.org.uk/
GTDB-TK	2.3.2	https://github.com.com/Ecogenomics/GTDBTk
Hifiasm	0.16.1-r375	https://github.com.com/chhylp123/hifiasm
HiGlass	1.11.6	https://github.com.com/higlass/higlass
MAGScoT	1.0.0	https://github.com.com/ikmb/MAGScoT
MaxBin	2.7	https://sourceforge.net/projects/maxbin/
MerquryFK	d00d98157618f4e8d1a9190026b19b471055b22e	https://github.com.com/thegenemyers/MERQURY.FK
MetaBat2	2.15-15-gd6ea400	https://bitbucket.org/berkeleylab/metabat/src/master/
MetaTOR	-	https://github.com.com/koszullab/metaTOR
MitoHiFi	2	https://github.com.com/marcelauliano/MitoHiFi
PretextView	0.2.5	https://github.com.com/wtsi-hpag/PretextView
PROKKA	1.14.5	https://github.com.com/vdejager/prokka
purge_dups	1.2.3	https://github.com.com/dfguan/purge_dups
Seqtk	1.3	https://github.com.com/lh3/seqtk
Singularity	3.9.0	https://github.com.com/sylabs/singularity
YaHS	yahs-1.1.91eebc2	https://github.com.com/c-zhou/yahs

***Genome annotation***

For annotation at the WSI, repeats were annotated using TETools 1.87 to softmask the assembly before predicting genes using BRAKER3 ( Stanke *et al.*, 2008). The protein data used was porifera proteins from UniProt, combined with protein sets from other ASG porifera predicted also with BRAKER3 to bootstrap the set. For transcriptome evidence 45 million semi-randomly sampled ( Stanke *et al.*, 2019) RNASeq spots from SRA were used. The gene set was also checked for completeness and contamination using Omark ( Nevers *et al.*, 2024).

Table 4 provides a list of relevant software tool versions and sources.

### Wellcome Sanger Institute – Legal and Governance

The materials that have contributed to this genome note have been supplied by a Tree of Life collaborator. The Wellcome Sanger Institute employs a process whereby due diligence is carried out proportionate to the nature of the materials themselves, and the circumstances under which they have been/are to be collected and provided for use. The purpose of this is to address and mitigate any potential legal and/or ethical implications of receipt and use of the materials as part of the research project, and to ensure that in doing so we align with best practice wherever possible. The overarching areas of consideration are:

•   Ethical review of provenance and sourcing of the material

•   Legality of collection, transfer and use (national and international)

Each transfer of samples is undertaken according to a Research Collaboration Agreement or Material Transfer Agreement entered into by the Tree of Life collaborator, Genome Research Limited (operating as the Wellcome Sanger Institute) and in some circumstances other Tree of Life collaborators.

## Data Availability

European Nucleotide Archive: *Petrosia ficiformis*. Accession number PRJEB56076; https://identifiers.org/ena.embl/PRJEB56076. The genome sequence is released openly for reuse. The *Petrosia ficiformis* genome sequencing initiative is part of the Aquatics Symbiosis Genomics (ASG) project. All raw sequence data and the assembly have been deposited in INSDC databases. Raw data and assembly accession identifiers are reported in Table 1.

## Author information

Contributors are listed at the following links:

- Wellcome Sanger Institute Tree of Life Management, Samples and Laboratory team
- Wellcome Sanger Institute Scientific Operations – Sequencing Operations
- Wellcome Sanger Institute Tree of Life Core Informatics team
- European Bioinformatics Institute ASG Data Portal team
- Wellcome Sanger Institute/Aquatic Symbiosis Genomics Project Leadership
