# Supplementary material for: The chromosomal genome sequence of the stone sponge Petrosia ficiformis (Poiret, 1789) and its associated microbial metagenome sequences
Source: Wellcome Open Res. 2025 Aug 18;10:450. [Version 1] doi: 10.12688/wellcomeopenres.24743.1 (PMC12648040; doi:10.12688/wellcomeopenres.24743.1)
Supplement: Peer review history

# Reviewer report: Reviewer response for version 1

**DOI:** 10.21956/wellcomeopenres.27260.r137178

**Published:** 2025-11-25

**Authors:** Robert Thacker

**Affiliations:** 1 Stony Brook University, Stony Brook, New York, USA

This manuscript presents a hologenome assembly of Petrosia ficiformis, adding to the recent set of sponge hologenomes assembled by the Wellcome Sanger Institute. Overall, the manuscript is well prepared, but some minor issues should be revised prior to publication.

 Background

 “These cyanobacteria are not obligate symbionts, since they are not vertically transmitted,…”

 This statement in the Background section confounds obligate vs. facultative associations with vertical vs. horizontal transmission. The mode of transmission can vary independently from the necessity of the association. The statement could be corrected by stating “These cyanobacteria are facultative symbionts and are not vertically transmitted…”

 This statement about reproduction needs additional context: “with spermatic cysts found in the mesohyl of males for no longer than 2.5 weeks.” Does this statement mean “than 2.5 weeks each year” or “than the 2.5 weeks prior to the release of sperm”? A citation is needed to support this statement.

 The statement “the population effectives of *P. ficiformis *appear to not rely on asexual reproduction” is confusing. What is meant by “effectives”?

 In the list of characteristics distinguishing S. spongiarum and S. feldmanni, the manuscript states: “(ii) *Ca. *S. feldmanni is located intracellularly, while *Ca. *S. spongiarum, extracellularly;” However, when describing the pocket bacteriocyte, the manuscript states “it hosts microbes extracellularly rather than intracellularly”. These two statements conflict and need to be reconciled, perhaps by stating “S. feldmanni is located in pocket bacteriocytes, while S. spongiarum occurs extracellularly in the mesohyl.”

 Later in this list of characteristics, the manuscript seems to misrepresent “leaky vertical transmission”, which refers to a combination of horizontal and vertical transmission. The statement could be corrected by stating “while *Ca. *S. spongiarum is transferred by both horizontal and vertical transmission (e.g, leaky vertical transmission).”

 Page 6 and Page 10 include identical paragraphs on Taxonomic Verification. This section is only needed on Page 6 and does not need to be repeated on Page 10.

Are sufficient details of methods and materials provided to allow replication by others?

Yes

Is the rationale for creating the dataset(s) clearly described?

Yes

Are the datasets clearly presented in a useable and accessible format?

Yes

Are the protocols appropriate and is the work technically sound?

Yes

Reviewer Expertise:

Sponge Ecology & Evolution, Microbial Symbioses

I confirm that I have read this submission and believe that I have an appropriate level of expertise to confirm that it is of an acceptable scientific standard, however I have significant reservations, as outlined above.

# Reviewer report: Reviewer response for version 1

**DOI:** 10.21956/wellcomeopenres.27260.r137172

**Published:** 2025-11-20

**Authors:** Cristina Díez-Vives

**Affiliations:** 1 National Centre for Biotechnology Department of Molecular and Cellular Biology, Madrid, Community of Madrid, Spain

The manuscript by Laura Steindler and colleagues describes the genome and metagenome sequencing of Petrosia ficiformis. The paper is well written and effectively highlights the relevance of this species for investigating both its genome and the genomes of associated microbiome. While I am not an expert in all the bioinformatic tools for the assembly, filtering, and annotation, the methods appear to be described in sufficient detail to allow reproducibility in future studies. I only have a couple of minor comments regarding specific details in the text.

 In the introduction, there is a reference to the 'leaky' transmission of Ca. S. spongiarum. However, Webster et al. 2010 describes a combination of vertical and horizontal transmission to explain the assemblage of entire sponge-associated microbial communities, rather than the transmission of a specific microbe. Moreover, the sentence “transferred vertically via ‘leaky’ transmission” is inaccurate. Individual microbes can be transmitted vertically, horizontally, or through mixed (so-called “leaky”) modes. Vertical transmission, by definition, would exclude horizontal transfer. The sentence should therefore read: “Ca. S. spongiarum is transmitted via ‘leaky’ transmission", implying the potential for both vertical and horizontal transfer”. More specific references could be: Schmitt et al. (2008), Vrijenhoek (2010), Ebert (2013), and Oliveira et al. (2020).

 Table 4. The source column has a mistake in most of the websites: "github.com.com/"

 WSI in Genome annotation has not been defined previously. 

 Why was the “squeeze” protocol from López et al. (2022) used in this study? Why is it necessary to separate the cellular interior from the mesohyl for genome sequencing? 

 The description of this protocol is itself somewhat ambiguous: it is referred to as a "squeeze enrichment of intact cells (eukaryotic and prokaryotic) from marine sponge tissues prior to routine DNA extraction", yet step 5 specifies “expelling the contents of the cellular interior into the L buffer.” The latter phrasing seems inconsistent with the stated goal of enriching intact cells, since expelling cellular contents implies cell disruption. Could the authors clarify why this specific protocol was chosen?

Are sufficient details of methods and materials provided to allow replication by others?

Yes

Is the rationale for creating the dataset(s) clearly described?

Yes

Are the datasets clearly presented in a useable and accessible format?

Yes

Are the protocols appropriate and is the work technically sound?

Yes

Reviewer Expertise:

microbial ecology, developmental biology, comparative metatranscriptomics

I confirm that I have read this submission and believe that I have an appropriate level of expertise to confirm that it is of an acceptable scientific standard.

# Reviewer report: Reviewer response for version 1

**DOI:** 10.21956/wellcomeopenres.27260.r131652

**Published:** 2025-09-23

**Authors:** Bernard M. Degnan

**Affiliations:** 1 The University of Queensland, St. Lucia, Queensland, Australia

The hologenome sequence of *Petrosia ficiformis *(Poiret, 1789) and its associated microbes is an important addition to the growing number of poriferan genomes. The Introduction provides a good introduction to the species and raises several important biological reasons for having this hologenome sequenced. The Methods and Results are sufficiently detail, with a few minor exceptions outlined below.

 Issues to address:

 Several abbreviations appear before they are fully named (e.g. MAGs, HMA and QV). Please correct.

 Is the phrase “…the population effectives...” in the Introduction correct?  Do you mean “effective population size”?

 Is “species *Candidatus* Synechococcus feldmanni” the correct nomenclature? Seems “Synechococcus feldmanni” should be italicised”.

 Figs 3 and 4 legends appear to be generic (e.g. there are no coloured bubbles in Fig. 3). Please check and correct if necessary.

 Some details appear incorrect or incomplete in two Methods statements:

1. “…sample was weighed and dissected on dry ice…” This is a sponge.  What is being dissected? Please provide details.
2. “…minced into small pieces using a scalpel and the cellular interior separated from the mesohyl using forceps”. What is the “cellular interior”? The mesohyl is the interior. And are these layers separated after mincing as written or before?  Please correct.
3. Following from this, from what part of the sponge was HMW DNA extracted?

Are sufficient details of methods and materials provided to allow replication by others?

Partly

Is the rationale for creating the dataset(s) clearly described?

Yes

Are the datasets clearly presented in a useable and accessible format?

Partly

Are the protocols appropriate and is the work technically sound?

Yes

Reviewer Expertise:

Marine biology and genomics; animals evolution and development

I confirm that I have read this submission and believe that I have an appropriate level of expertise to confirm that it is of an acceptable scientific standard, however I have significant reservations, as outlined above.
